# Aristotle’s Illusion in Parkinson’s Disease: Evidence for Normal Interdigit Tactile Perception

**DOI:** 10.1371/journal.pone.0088686

**Published:** 2014-02-11

**Authors:** Mirta Fiorio, Angela Marotta, Sarah Ottaviani, Lara Pozzer, Michele Tinazzi

**Affiliations:** 1 Department of Neurological and Movement Sciences, University of Verona, Verona, Italy; 2 Neurology Unit and Department of Neuroscience, Borgo Trento Civil Hospital, Verona, Italy; University of Reading, United Kingdom

## Abstract

Sensory alterations, a common feature of such movement disorders as Parkinson’s disease (PD) and dystonia, could emerge as epiphenomena of basal ganglia dysfunction. Recently, we found a selective reduction of tactile perception (Aristotle’s illusion, the illusory doubling sensation of one object when touched with crossed fingers) in the affected hand of patients with focal hand dystonia. This suggests that reduced tactile illusion might be a specific feature of this type of dystonia and could be due to abnormal somatosensory cortical activation. The aim of the current study was to investigate whether Aristotle’s illusion is reduced in the affected hand of patients with PD. We tested 15 PD patients, in whom motor symptoms were mainly localised to one side of the body, and 15 healthy controls. Three pairs of fingers were tested in crossed (evoking the illusion) or parallel position (not evoking the illusion). A sphere was placed in the contact point between the two fingers and the blindfolded participants had to say whether they felt one or two stimuli. Stimuli were applied on the affected and less or unaffected side of the PD patients. We found no difference in illusory perception between the PD patients and the controls, nor between the more affected and less/unaffected side, suggesting that Aristotle’s illusion is preserved in PD. The retained tactile illusion in PD and its reduction in focal hand dystonia suggest that the basal ganglia, which are dysfunctional in both PD and dystonia, may not be causally involved in this function. Instead, the level of activation between digits in the somatosensory cortex may be more directly involved. Finally, the similar percentage of illusion in the more affected and less or unaffected body sides indicates that the illusory perception is not influenced by the presence or amount of motor symptoms.

## Introduction

Movement disorders, such as Parkinson’s disease (PD) and dystonia, are characterised not only by motor symptoms but also by somatosensory alterations [1]–[6]. For instance, patients with PD display a number of somatosensory deficits, including abnormal temporal [4], [7], [8] and spatial discrimination of sensory stimuli [2], [9] and altered proprioception [10]–[14]. Similar abnormalities in sensory discrimination [15]–[25], as well as in proprioceptive functions [26]–[32], have also been observed in primary dystonia. These abnormalities are not restricted to a specific type of dystonia; they can also emerge in patients with blepharospasm, focal hand, cervical, and generalized dystonia. Moreover, sensory deficits in dystonia are not strictly related to the affected limb but can also occur in body parts remote from dystonic symptoms [19], [24], [31], [33]. This evidence challenges the notion of specificity of sensory abnormalities to the pathophysiology of different movement disorders [23]. The presence of similar sensory deficits in different kinds of movement disorders seems to be an epiphenomenon related to a common underlying factor: a dysfunction of the basal ganglia [4], [23]. These subcortical structures preside over movement control and somatosensory processing [34], such as temporal and spatial discrimination of tactile stimuli [35], [36].

Recently, tactile perception has been explored in dystonic patients by applying a tactile illusion paradigm: the so-called Aristotle illusion [37]. This paradigm allows to investigate interdigit functional somatosensory relations, by applying a single stimulus at the contact point of two crossed or parallel fingertips and then measuring the percentage of illusory doubling perception in the blindfolded subject [38], [39]. Typically, illusory doubling occurs in the crossed fingers position, whereas the realistic perception of the single stimulus is usually associated with the parallel fingers position [37], [39], [40]. By investigating this tactile illusion in different digit pairs of the dominant and the non-dominant hand, we observed a very specific alteration only in the affected hand of patients with focal hand dystonia but not in those with other types of dystonia, such as cervical dystonia and blepharospasm [37]. This sensory abnormality correlated with disease severity, hinting at a relation between the motor symptoms in focal hand dystonia and this specific type of tactile function.

The aim of the present study was to determine whether this tactile function is differently affected in PD, another common basal ganglia-related movement disorder. To do this, we selected PD patients in whom the motor symptoms were prevalently localised to one side of the body. By comparing the illusory doubling perception in the more affected and the less or unaffected hand of the PD patients, we wanted to disentangle the role motor symptoms may play in this kind of tactile perception.

## Materials and Methods

### Participants

Fifteen right-handed patients with Parkinson’s disease (PD) (11 males; mean age ± standard deviation, 61.33±8.13 years) and fifteen healthy controls (6 males; mean age, 61.73±6.86 years) participated in the study.

Exclusion criteria for PD patients were: tremor at the arm and peripheral sensory impairment as evidenced from EMG recording. The inclusion criterion was a Hoehn & Yahr score (H&Y) [41] ≤ 2, indicating that the motor symptoms were mostly localised to one side of the body. Nine patients were prevalently (N = 6) or exclusively (N = 3) affected on the right side, and six patients were prevalently (N = 5) or exclusively (N = 1) affected on the left side. The mean disease duration was 4.82±3.30 years (range, 1–11 years). Disease severity at the motor examination of the Unified Parkinson’s Disease Rating Scale part III [42] was 12.27±3.65 (range, 8–20) in the off-state. In order to avoid the confounding factor of treatment, the PD patients were tested after overnight withdrawal of medication (i.e., L-dopa or other dopaminergic drugs) and the washout period was at least 12 h for each patient. Table 1 reports the demographic and clinical characteristics of the patients.

**Table 1:** Demographic and clinical data of patients.

Patient	Sex	Age (years)	Affected side	Disease duration (years)	UPDRS^a^	H&Y^b^
1	M	65	Right	3	14	2
2	M	72	Right	10	18	2
3	M	60	Right	2	9	1.5
4	M	65	Right	7	14	2
5	M	77	Right	1	8	1
6	M	50	Right	5	8	1.5
7	M	63	Left	4	13	1.5
8	F	65	Right	6	12	2
9	F	59	Right	10	10	2
10	F	52	Right	11	8	2
11	M	63	Left	6	12	2
12	M	51	Left	5	20	2
13	M	56	Left	6	11	2
14	F	70	Left	1	11	2
15	M	52	Left	3	16	2

a  =  Unified Parkinson’s Disease Rating Scale – III part (motor evaluation).

b  =  Hoehn & Yahr staging scale.

All study participants were informed about the experimental procedures and provided written informed consent before taking part in the study. The ethical committee of the Department of Neurological and Movement Sciences, University of Verona, Italy, approved the study design and protocol.

### Procedure

Aristotle’s illusion refers to the illusory perception of two objects when a small object is placed in the contact point between two crossed fingertips [43]. We recently applied this paradigm in patients with dystonia [37]. In order to investigate the same tactile function also in patients with Parkinson’s disease, we followed as much as possible the very same procedure adopted in our previous study [37]. In particular, we applied for 5 seconds one or two spheres on the fingertips of three digit pairs: second-third (D2-D3); second-fourth (D2-D4); and fourth-fifth (D4-D5). The single sphere (8 mm in diameter) and the two spheres (each 4 mm in diameter) were attached to a von Frey filament in order to apply the same pressure (about 35 g) during the task.

**Figure 1:**
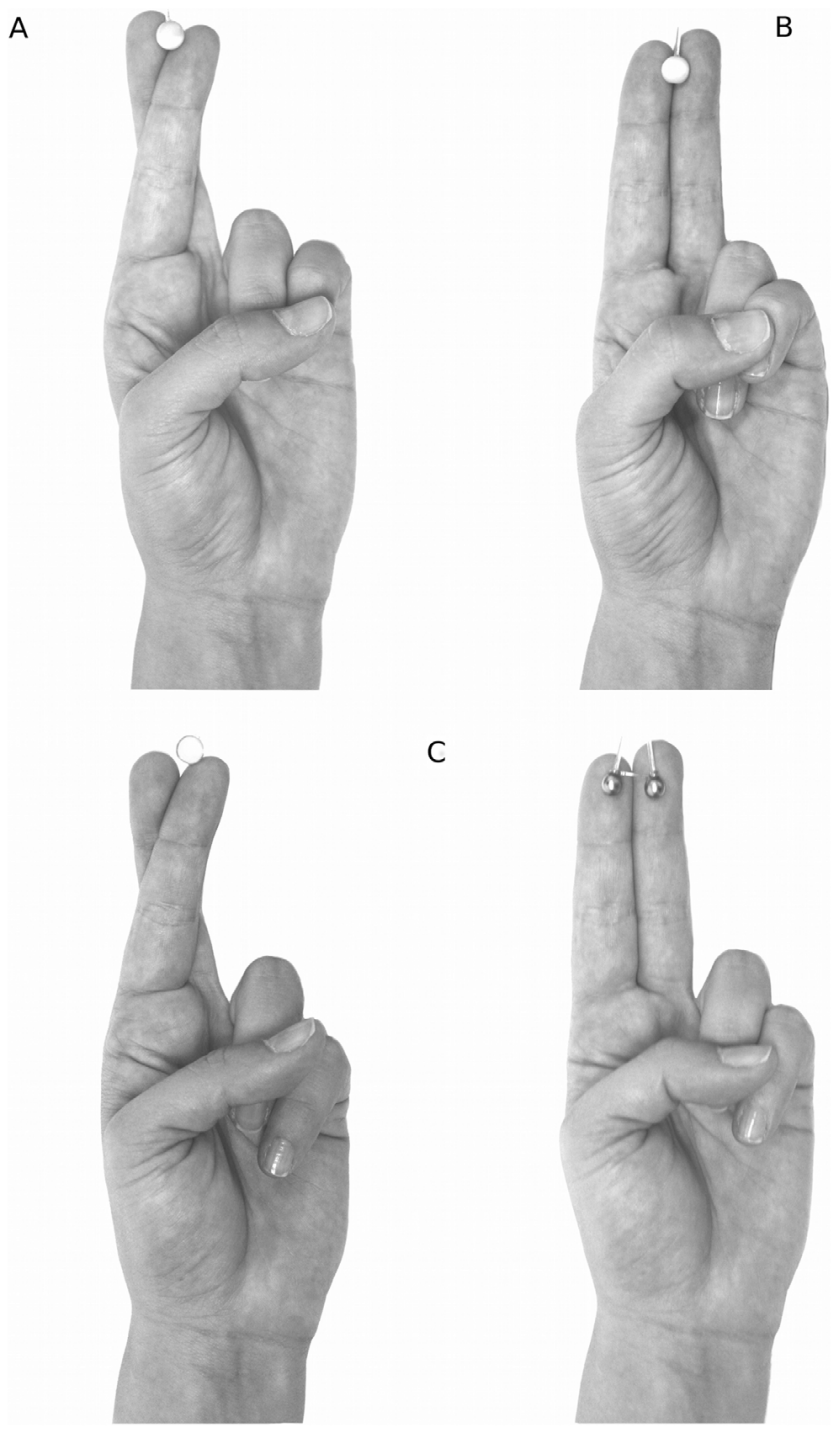
Experimental and control conditions. The experimenter set and maintained the correct position of the subject’s fingers during stimulation. A) Experimental condition: the digits were crossed and a sphere was placed in the contact point between them. This condition is usually associated with Aristotle’s illusion. B) Control condition: the digits were placed parallel and a sphere was placed in the contact point between them. This condition is usually associated with correct perception of an object. C) The additional control conditions for the crossed (*left*) and parallel (*right*) finger positions. In the first, the fingers were crossed and one sphere was placed on one finger, near the contact point. In this way it was possible to exclude an association between the crossed position and the “two-stimuli” response. In the second condition, the fingers were parallel and two spheres were simultaneously placed on them. This condition avoided the association between the parallel position and the “one-stimulus” response.

The participants were blindfolded and seated in a chair with one hand placed palm up on the table. The experimenter asked the participants to say how many stimuli they perceived on their fingertips. The stimuli could not be seen until the end of experiment. The subjects were briefly trained to familiarize them with the task. In the experimental condition that usually evokes the illusion, one sphere was placed in the contact point between two crossed fingers (Figure 1A). As a control condition, the same sphere was placed in the contact point between two parallel fingers (Figure 1B). In order to avoid any response bias due to a simple association with the finger position (crossed position – “two stimuli”, parallel position – “one stimulus”), two other control conditions were set up. One entailed crossing the subject’s fingers and placing a single sphere only on one finger, near the contact point (Control1). Another control condition involved simultaneous placement of two spheres on two parallel fingers (Control2) (Figure 1C). All the digit pairs were passively moved and kept into contact by the experimenter until the participant’s response. This procedure allowed the participants to maintain the fingers in crossed or parallel position even for the most difficult configurations (i.e., D2-D4). We tested both sides in the PD patients and the left and right hands in the healthy controls. On each side, we tested all the conditions (crossed, parallel, Control1 and Control2) in one single session for a total of 90 trials: 30 trials in the crossed condition (10 trials for each digit pair); 30 trials in the parallel condition (10 trials for each digit pair); 15 trials in the control condition with crossed fingers, Control1 (5 trials for each digit pair) and 15 trials in the control condition with parallel fingers, Control2 (5 trials for each digit pair). We computed the percentage of the “two stimuli” response in each finger position (crossed and parallel) and finger pair (D2-D3, D2-D4, D4-D5) as an index of illusion.

### Statistical analyses

Preliminary analyses to verify that patients and healthy controls were comparable for age and gender were performed using Student’s t-test and the chi-square test, respectively. Furthermore, because the PD group included some patients more affected on the right side and others more affected on the left side, we had to exclude that the perception of the illusion in the control group was related to the tested hand. Therefore, the amount of illusion in the left and the right hands of the controls was compared by means of t-tests separately for each condition (crossed and parallel position) and finger pair (D2-D3, D2-D4, D4-D5).

In order to compare the amount of illusion in the two groups, we performed a repeated measures analyses of variance (ANOVA) with group (PD patients vs. Controls) as the between-subjects factor and finger position (crossed vs. parallel), finger pair (D2-D3, D2-D4, D4-D5) and body side (affected vs. less/unaffected) as the within-subjects factors. Bonferroni correction for multiple comparisons was used when necessary. Finally, in order to exclude peripheral deficits in tactile perception, we compared the percentage of correct response, computed as average across all the digit pairs, in the additional control conditions (Control1: crossed fingers, one sphere on one fingertip; and Control2: parallel fingers, two spheres simultaneously placed on the two fingertips) between PD patients and controls by means of repeated measures analysis of variance (ANOVA). For each hand, we considered as between-subjects factor the group (PD patients vs. Controls) and as within-subjects factor the control condition (Control1-crossed vs. Control2-parallel). Statistical significance was set at P<0.05.

## Results

Preliminary analyses of the demographic characteristics confirmed that the two groups were comparable for age [t (28)  = –0.146, P = 0.885] and gender distribution [χ^2^ = 3.394, P = 0.065]. In the control group there was a non-significant difference in the “two stimuli” response between the left and the right hands in both the crossed and parallel conditions (for all digit pairs, –0.564<t (14) <0.774, P>0.45). Hence, we were able to consistently compare the right hand of the controls with the affected hand in the PD patients and the left hand of the controls with the less/unaffected hand of the PD patients.

**Figure 2:**
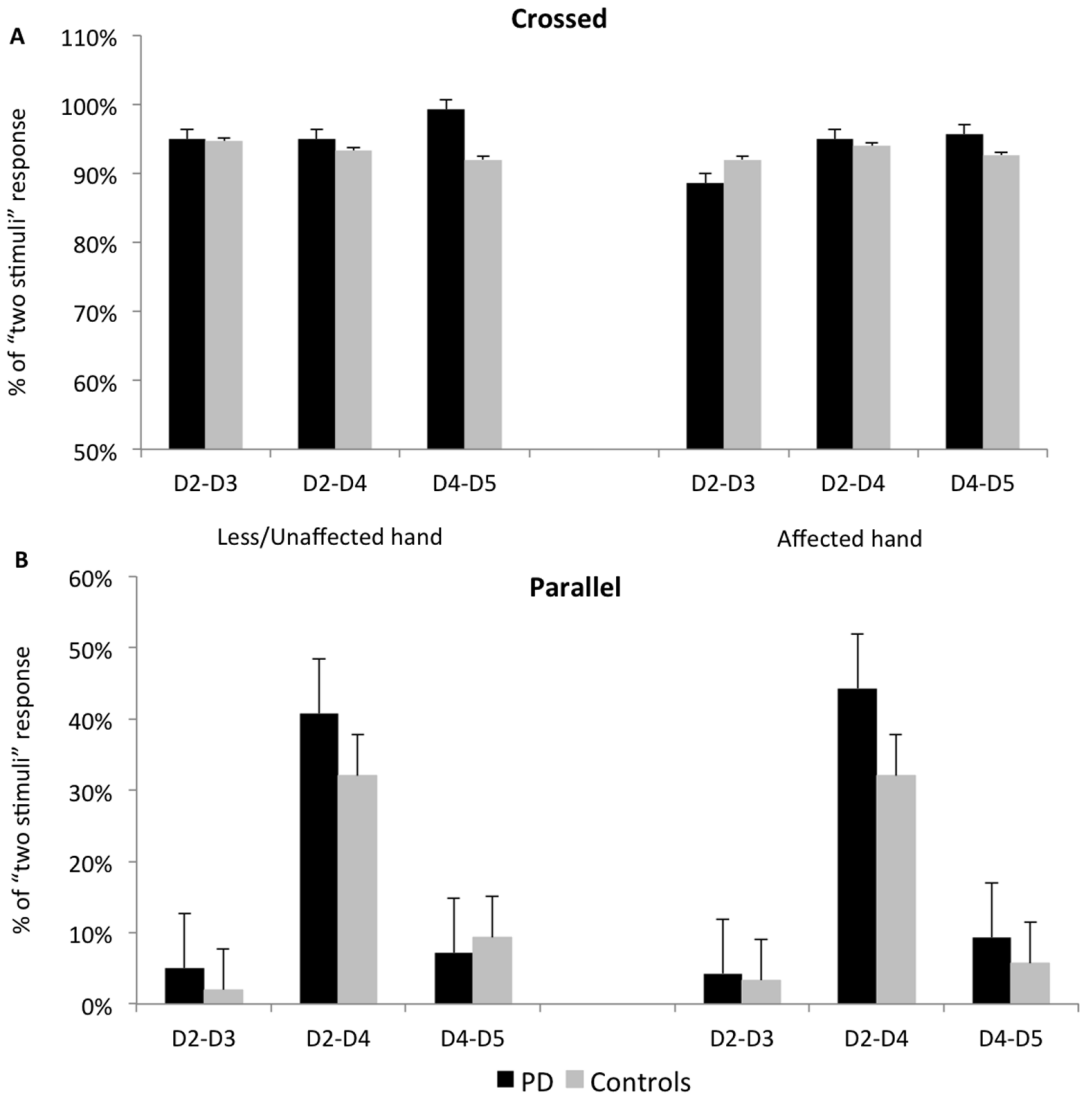
Aristotle’s illusion in patients with Parkinson’s disease (PD) versus healthy controls. The columns represent the mean percentage of illusion perceived in all the tested digit-pairs of both the affected and the less/unaffected hand. The bars represent the standard error. In the crossed position (A) the patients perceived the illusion like the controls. There were no differences between the two groups also in the parallel finger position (B). Hence, contrary to other aspects of somatosensory perception, Aristotle’s illusion is preserved in PD.

As expected, the main analysis showed that the finger position factor was significant (F (1,28)  = 435.2, P<0.001). This was due to an overall higher percentage of the “two stimuli” response in the crossed (mean ± standard error of the mean, 93±2.3%) than in the parallel finger position (16.7±3.3%) (Figure 2A, B). The finger pair factor was also significant (F (2,56)  = 21.3, P<0.001), due to a higher percentage of the “two stimuli” response in the finger pair D2-D4 (65.3±3.9%) compared to D2-D3 (47.2±1.9%, P<0.001) and to D4-D5 (52±2%, P = 0.001). Moreover, the interaction finger position × finger pair was also significant (F (2,56) = 18.3, P<0.001). Post-hoc comparisons showed that in the parallel finger position the percentage of the “two stimuli” response was higher in the finger pair D2-D4 (37.5±6.7%) compared to D2-D3 (3.5±1.4%, P<0.001) and D4-D5 (9.2±3.5%, P<0.001) (Figure 2B)”. The effects of group and body side and all the other interactions between the factors were not significant (P>0.333); therefore, the patients perceived the illusion like the healthy controls, independently of whether the stimulus was applied to the affected or the unaffected side.

**Figure 3:**
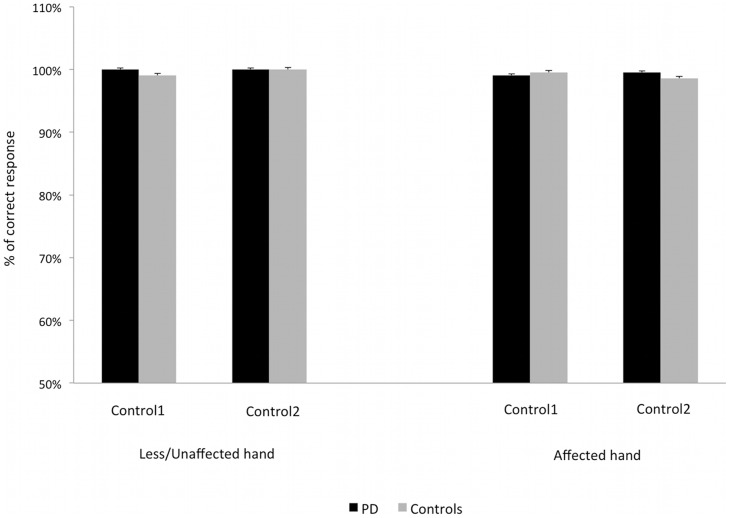
Additional control conditions in PD patients and healthy controls. The columns represent the mean percentage of correct response averaged across all the digit pairs for the affected and the less/unaffected hand. Control1 refers to the control condition with crossed fingers and one sphere on one fingertip, Control2 refers to the control condition with parallel fingers and two spheres simultaneously placed on the two fingertips. The bars represent the standard error. The two groups showed high and comparable number of correct responses in each additional control condition.

Finally, the analysis of the additional control conditions on both hands did not reveal significant effect of group (affected hand: F (1,28)  = 0.18; P = 0.679; less/unaffected hand: F (1,28)  = 2.15, P = 0.153), or control condition (affected hand: F (1,28)  = 0.14, P = 0.711; less/unaffected hand: F (1,28)  = 2.15, P = 0.153). Furthermore, the interaction group*control condition was not significant (affected hand: F (1,28)  = 1.26, P = 0.271; less/unaffected hand: F (1,28)  = 2.15, P = 0.153). Notably, the fact that the percentage of correct response was overall high in both the Control1-crossed condition (99.4%±0.02) and Control2-parallel condition (99.6%±0.2%), confirms that the finger position per se does not affect tactile perception (Figure 3).

## Discussion

This study investigated tactile perception in Parkinson’s disease patients by means of Aristotle’s illusion. In line with previous studies [37]–[39], [40], [44], the illusory doubling of one tactile stimulus was perceived more frequently in the crossed than in the parallel finger position, thus confirming the consistency of the applied paradigm. Aristotle’s illusion arises because the rules of tactile exploration are subverted. Namely, we commonly explore objects by keeping our fingers in a parallel position. On the basis of this repeated tactile experience, a relation between proprioceptive (finger position) and tactile information is built. Proprioceptive information is associated with the way in which the brain processes the tactile information derived from an object: the sensory signals simultaneously coming from two adjacent skin areas are integrated in a unique percept by the brain, whereas the sensory signals coming from two non-adjacent skin areas are usually kept separate [39].

In Aristotle’s illusion, the fingers are crossed and the tactile signals are detected from two usually distant skin areas. This unusual finger configuration violates the rules of perception: skin areas brought into contact are usually distant from one another and most frequently touched by two objects. Because the brain is not fast enough to readapt the frame of reference to the new finger configuration, the sensory signals are processed as if the crossed fingers were in a parallel position [44]. In first instance, the brain solves the conflict between the incoming proprioceptive and the tactile information by means of the illusion. This physiological mechanism clearly appeared also in our sample. In all the digit pairs of both hands, the amount of illusion was higher in the crossed than in the parallel finger position.

The illusory doubling sensation occurred more frequently in the parallel position of digits D2-D4 than in the other digit pairs (D2-D3 and D4-D5). This finding is in line with previous studies [37], [40] and underscores the concept that the distance between the touched skin areas influences the tactile perception of objects. Indeed, D2 and D4 are non-adjacent digits and their functional relation is lower than that of adjacent digit pairs. Hence, the sensory signals simultaneously deriving from D2-D4 may not be well integrated, thus evoking the illusory perception of two stimuli even when the fingers are in a parallel position. Conversely, the sensory signals from adjacent and more functional related digit pairs (like D2-D3 and D4-D5) are more frequently elaborated in an integrated manner, resulting in the realistic perception of a single stimulus applied to the contact point of the parallel fingers. Summarizing, the simultaneous stimulation with one sphere of D2-D4 in parallel position evoked the illusory doubling perception more frequently than the other digit pair because of the less functional relation existing between these two non-adjacent fingers [40].

We found no significant difference in illusory perception between the two groups. This result hints at some main considerations: i) the same percentage of illusion in PD patients and control subjects suggests that the basal ganglia, which are mainly impaired in PD, are not directly involved in the tactile function related to Aristotle’s illusion; ii) the same amount of illusion in both the more affected and the less or unaffected hand of PD patients suggests that the presence of motor symptoms does not influence the illusory perception.

By unveiling a different tactile illusory perception between focal hand dystonia, in which it is reduced [37], and non-hand dystonia [37] and PD (current study), in which it is preserved, Aristotle’s illusion might help to shed new light on the mechanisms underlying sensory alterations in different movement disorders. More precisely, the specificity of reduced Aristotle’s illusion in focal hand dystonia [37] argues against a prominent role of the basal ganglia and hints instead at a major involvement of cortical mechanisms. This hypothesis is consistent with observations reported in a recent neurophysiological study investigating the neural correlates of Aristotle’s illusion in healthy subjects [44]. As revealed in that study and as found in previous investigations on other types of tactile illusions [45], [46], activity in the primary somatosensory cortex reflects the illusory doubling perception rather than the physical characteristics of the tactile stimuli.

The localization of tactile stimuli initially engages a somatotopic reference frame, reflecting the canonical posture of the body. Subsequently, the proprioceptive information about the real position of the body is integrated [47]. The first part of this process occurs in the primary somatosensory cortex and explains Aristotle’s illusion [44]. Hence, it is plausible that a specific alteration in finger representation could undermine this specific type of tactile illusion. The fact that the illusion is altered in focal hand dystonia [37], but not in PD, supports the main role of somatosensory cortical alterations in focal hand dystonia and not in PD. Indeed, it has been widely demonstrated that finger representation in the primary somatosensory cortex is abnormal in focal hand dystonia [48]–[50].

In our previous study we interpreted the reduction of Aristotle’s illusion in focal hand dystonia as a behavioural consequence of alterations in the level of cortical activation related to tactile stimulation [37]. With regard to the current study, it is reasonable to assume that the functional activation of finger representation in the somatosensory cortex is retained in PD patients. Summarizing, the fact that Aristotle’s illusion is altered only in focal hand dystonia [37] but is preserved in PD patients suggests that this tactile illusion requires cortical regions, i.e., the primary somatosensory cortex, rather than subcortical structures like the basal ganglia.

For this study we recruited patients at an early and middle stage of disease in which motor symptoms were mainly localised to one side of the body. This choice allowed us to compare the amount of sensory illusion between the more affected and the less or unaffected limb. It could be argued, however, that no sensory alterations are clearly detectable at this stage of disease. According to a recent fMRI study, changes in brain activity and connectivity can occur in early PD though such patients may demonstrate normal performance on a tactile task [51]. Although we cannot exclude that PD patients in advanced or late stage of disease could display abnormalities at Aristotle’s illusion paradigm, we find this prediction quite unlikely, since no study to date has proved the existence of an unbalanced level of activation between digits in PD that could account for a reduced illusory doubling perception. Moreover, somatosensory alterations, such as temporal discrimination of tactile [7] and proprioceptive [52] stimuli, have been found even in samples of PD patients at early and mild stages of disease, confirming that the absence of sensory deficits at Aristotle’s illusion task may not be related to the early stage of disease. Furthermore, alterations at spatial discrimination tests are well documented in PD [1], [2], [9], [53], [54]. Independently from the task used to measure the spatial discrimination acuity (i.e., two-point discrimination or grating orientation task) all these studies reported increased spatial discrimination threshold in PD patients compared to control subjects. In particular, a study by Shin and colleagues [9] in a group of patients with clinical characteristics similar to our sample (such as the early stage of disease), demonstrated that PD patients off-medication had significantly higher sensory thresholds on the palmar surface of the tips compared to healthy control. Despite these clear tactile deficits in PD patients, based on our findings we could hypothesize that the tactile processing associated to the functional relation between fingertips (involved in Aristotle’s illusion) is spared in PD. Hence, we could speculate that the dissociation between impaired (i.e., spatial and temporal discrimination) and preserved (i.e., Aristotle’s illusion) tactile functions in PD may be related to the different involvement of the basal ganglia and the primary somatosensory cortex respectively in the pathophysiology of PD. This speculation, however, is derived from the extensive literature on sensory alterations in PD, since we did not directly test other sensory functions in our PD patients, apart from Aristotle’s illusion.

The retained tactile illusory perception in both the more affected and the less or unaffected hand of the PD patients suggests that the mere presence of motor symptoms is insufficient to reduce this tactile function and that the illusory perception is independent of the pathophysiology of the movement disorder.

## Conclusion

Our study demonstrates that the functional relation between fingers during tactile perception is preserved in PD patients; furthermore, this finding corroborates our observations in a previous study in patients with focal hand dystonia [37]. Taken together, our results suggest that an alteration in the functional relation between fingers in tactile perception is a distinctive feature of focal hand dystonia and not of other movement disorders. Finally, in keeping with a recent neurophysiological study [44], the mechanisms of Aristotle’s illusion may be mainly related to activity in the primary somatosensory cortex rather than in the basal ganglia.
